# Examining the association between rurality and positive childhood experiences among a national sample

**DOI:** 10.1111/jrh.12708

**Published:** 2022-08-27

**Authors:** Elizabeth Crouch, Janice C. Probst, Sylvia Shi, Alexander McLain, Jan M. Eberth, Monique J. Brown, Melinda Merrell, Kevin J. Bennett

**Affiliations:** ^1^ Rural and Minority Health Research Center, Arnold School of Public Health University of South Carolina Columbia South Carolina USA; ^2^ Department of Health Services Policy & Management, Arnold School of Public Health University of South Carolina Columbia South Carolina USA; ^3^ Department of Epidemiology and Biostatistics, Arnold School of Public Health University of South Carolina Columbia South Carolina USA; ^4^ Department of Family and Preventive Medicine, School of Medicine University of South Carolina Columbia South Carolina USA

**Keywords:** mental health, positive childhood experiences, resilience, rural, trauma

## Abstract

**Purpose:**

The present study examines the association between rurality and positive childhood experiences (PCEs) among children and adolescents across all 50 states and the District of Columbia. Recent work has quantified the prevalence of PCEs at the national level, but these studies have been based on public use data files, which lack rurality information for 19 states.

**Methods:**

Data for this cross‐sectional analysis were drawn from 2016 to 2018 National Survey of Children's Health (NSCH), using the full data set with restricted geographic data (n = 63,000). Descriptive statistics and bivariate analyses were used to calculate proportions and unadjusted associations. Multivariable regression models were used to examine the association between residence and the PCEs that were significant in the bivariate analyses.

**Findings:**

Rural children were more likely than urban children to be reported as having PCEs: volunteering in their community (aOR 1.29; 95% CI 1.18‐1.42), having a guiding mentor (aOR 1.75; 95% CI 1.45‐2.10), residing in a safe neighborhood (aOR 1.97; 95% CI 1.54‐2.53), and residing in a supportive neighborhood (aOR 1.10; 95% CI 1.01‐1.20) than urban children.

**Conclusions:**

The assessment of rural‐urban differences in PCEs using the full NSCH is a unique opportunity to quantify exposure to PCEs. Given the higher baseline rate of PCEs in rural than urban children, programs to increase opportunities for PCEs in urban communities are warranted. Future research should delve further into whether these PCEs translate to better mental health outcomes in rural children.

## INTRODUCTION

Research in childhood trauma has begun to examine not only adverse childhood experiences (ACEs), which encompass experiences of household dysfunction, neglect, and abuse, but also positive childhood experiences (PCEs), which include safe and supportive environments for children to grow and learn for healthy social‐emotional development.^1^, ^2^ Both types of experiences have been found to influence physical and mental health outcomes for children and adolescents into adulthood.^3^, ^4^ While ACEs have been associated with riskier behaviors and poorer outcomes, PCEs have been shown to help reduce the effects of ACEs and build resilience in rural and urban communities.^5^, ^6^

While ACEs may mostly occur inside the home, PCEs are often provided in community settings, such as schools or churches.^7^ Prior work has focused on the higher rates of ACE exposure in rural areas, with rural children and adolescents having higher rates of exposure to parental separation/divorce, parental death, household incarceration, household violence, household mental illness, and household substance abuse than their urban peers.^8^ The National Advisory Committee on Health and Human Services states that the prevention and mitigation of ACEs is one of their priority areas.^9^

One way to prevent, mitigate, and build resilience among children is through PCEs, which have been best described through the Healthy Outcomes Positive Experiences framework.^2^ This framework categorizes PCEs into 4 categories: (1) nurturing, supportive relationships, (2) safe, stable environments, (3) constructive social engagement, and (4) development of social and emotional competencies.^2^ PCEs have been previously shown to enhance healthy social‐emotional development in children.^10^, ^11^, ^12^

Recent work has begun to quantify the prevalence of PCEs at the national level, but these studies have been based on public use data files, which lack rurality information for 19 states.^8^, ^13^ While this early work found that rural children had a higher likelihood of experiencing community service or volunteer work, school, or faith‐based organizations and having a mentor for guidance, compared to their urban counterparts,^8^ it is unclear whether these findings would hold true for a sample that includes all 50 states. The present study examines the association between rurality and PCEs among children and adolescents across all 50 states and the District of Columbia, while also including adjusted analyses to highlight rural‐influenced outcomes. Examining the factors that are associated with exposure to PCEs may be helpful to policymakers and stakeholders as they design interventions for children and adolescents in rural communities.

## METHODS

### Data source

Data for this cross‐sectional analysis were drawn from 2016 to 2018 National Survey of Children's Health (NSCH). The NSCH is a combined online and mail survey, asking caregivers of children and adolescents (up to 17 years) about child health.^14^ Because detailed address information is not available in the public use data set, we used NSCH restricted data sets at the Triangle Research Data Center (RDC) in Raleigh, NC. While data access through an RDC allowed complete identification of rural versus urban residence, it came with restrictions designed to prevent inadvertent data disclosure. These are more fully discussed below.

### Population studied

The 2016‐2018 NSCH included 102,341 children, with 50,212 interviews in 2016, 21,599 in 2017, and 30,530 in 2018. We restricted our sample to school‐age children (age 6 and older) who would have been of age to experience the PCEs measured. The sample was further restricted to respondents with complete demographic information, ACE, and PCE questions. The final unweighted data sample used for this study was approximately 63,000 children. Data were subject to rounding to meet RDC disclosure restrictions.

### Construction of primary outcome of interest and covariates

There were 7 PCEs constructed based on prior literature^8^, ^15^ and the 4 categories of PCEs specified by Sege and Brown.^2^ PCEs were defined as the reported presence in the child's life of 7 specific experiences: after‐school activities, community volunteer, guiding mentor, connected caregiver, safe neighborhood, supportive neighborhood, and resilient family. Specific NSCH questions used to establish these factors are listed in Table 1.

**TABLE 1:** NSCH questions used to identify positive childhood experiences

Concept	Questions
After‐school activities	During the past 12 months, did this child participate in any organized activities or lessons, after school or on weekend?
Community volunteer	During the past 12 months, did this child participate in any type of community service or volunteer work at school, church, or in the community?
Guiding mentor	Other than you or other adults in your home, is there at least 1 other adult in this child's school, neighborhood, or community who knows this child well and who he or she can rely on for advice or guidance?
Connected caregiver	How well can you and this child share ideas or talk about things that really matter?
Safe neighborhood	To what extent do you agree [that] the child is safe in [your] neighborhood?
Supportive neighborhood	To what extent do you agree [that] people in this neighborhood help each other out, we watch out for each other's children in this neighborhood, when we encounter difficulties, we know where to go for help in our community?
Resilient family	When your family faces problems, how often are you likely to do each of the following? Stay hopeful even in difficult times Work together to solve our problems

Covariates were selected based on Andersen's Behavioral Model.^16^ Demographic characteristics of the child included sex, age, race/ethnicity, and special health care needs of the child. Age was grouped into 6‐12 years of age, and 13‐17 years of age, with the categories chosen based on childhood versus adolescence. Race/ethnicity, conceptualized as a measure for possible exposure to discrimination, was categorized as non‐Hispanic white, non‐Hispanic Black, Hispanic, American Indian/Alaska Native, Asian/Pacific Islander, and “Other” racial groups. Special health care needs were codified using the NSCH 5‐item indicator tool that asks about prescription medicine, use of services, functional mobility, therapy, and ongoing conditions (physical, emotional, developmental, and behavioral).

Household and caregiver characteristics included residence (rural or urban), respondent relation to the child, primary language spoken in the home, educational attainment of the caregiver, family structure, or poverty/income level. Rural‐urban status was determined at the census tract level using the 2013 Rural‐Urban Commuting Area codes,^17^ with codes 1‐3 categorized as urban and codes 4‐10 considered rural. Relations with the child included mother, father, and other. The primary language in the home was coded as English or not English. Caregiver educational attainment was dichotomized into those with less than or equal to high school/GED and those with at least some college education or more. The family structure had 4 groups: 2 parents, currently married; 2 parents, not currently married; a single mother; and other. Poverty/income level had 4 levels: 0%‐99% of the federal poverty level (FPL), 100%‐199% FPL, 200%‐399% FPL, and 400% FPL or above.

### Analytic methods

Descriptive statistics and bivariate analyses were used to calculate proportions and unadjusted associations. Multivariable regression models were used to examine the association between residence and the PCEs that were significant in the bivariate analyses. The survey sampling weights, cluster, and strata that were constructed by the NSCH were used to ensure accurate proportions and model estimates. Further information on the NSCH sampling plan can be found on the DRC website. SAS (SAS version 9.3; SAS Institute Inc., Cary, NC) was used for all analyses. As noted above, confidentiality requirements restricted the output we were allowed to present. Thus, we can present estimated percentages for population characteristics but are not allowed to present the standard errors associated with those estimates.

### Ethical considerations

The study was approved as exempt by the [name concealed for review] Institutional Review Board.

## RESULTS

### Characteristics of studied children and adolescents

Nearly, 12% of our sample resided in a rural area (11.7%, Table 2). Over 50% of the children in our sample were male (51.3%), aged 6‐12 years (58.3%), and non‐Hispanic white (52.9%). The majority of children had private insurance (59.9%), a caregiver with at least some college education or beyond (71.7%), and lived with both parents who are currently married (66.7%). Less than a quarter of children with special health care needs (23.1%). The primary respondent for the child was the mother (62.4%). Just over 13% of children had a primary language other than English (13.4%). Nearly, a fifth of the children in our sample lived in a household with resources below the FPL (19.1%).

**TABLE 2:** Characteristics of children ages 6‐17, National Survey of Children's Health (years), in total and stratified by residence

	All (%)	Rural	Urban	*P* value
Characteristic		%	%	
*Characteristics of child*				
Sex of child				.1037
Male	51.3	52.8	51.1	
Female	48.7	47.2	48.9	
Age of child				.3113
6‐12 years old	58.3	57.4	58.4	
13‐17 years old	41.7	42.6	41.6	
Race/ethnicity of child				<.0001
Non‐Hispanic white	52.9	74.4	50.1	
Non‐Hispanic African American	13.1	8.1	13.8	
Hispanic	24.5	11.4	26.2	
NH American Indian/Alaska Native	4.4	0.8	4.8	
NH Asian/Pacific Islander	0.4	0.8	0.3	
Other	4.7	4.4	4.7	
Special health care needs				.1396
Yes	23.1	24.2	23.0	
*Characteristics of parent/household*				
Respondent's relation to child				<.0001
Mother	62.4	64.8	62.1	
Father	27.3	22.5	28.0	
Other	10.3	12.7	10.0	
Primary language				<.0001
Not English	13.4	6.3	14.4	
Guardian education				<.0001
Less than high school or high school	28.3	35.0	27.5	
Some college or more	71.7	65.0	72.5	
Family structure				.0002
Two parents, currently married	66.7	64.9	67.0	
Two parents, not currently married	7.7	7.0	7.8	
Single mother	19.4	20.2	19.3	
Other	6.1	8.0	5.9	
Poverty/income level				<.0001
0%‐99% federal poverty level	19.1	23.0	18.6	
100%‐199% federal poverty level	21.3	25.0	20.8	
200%‐399% federal poverty level	27.4	32.4	26.8	
400% federal poverty level or above	32.1	19.6	33.8	
Health insurance for child				<.0001
Public	28.8	35.0	28.0	
Private	59.9	50.4	61.2	
Public and private	4.4	5.7	4.2	
Not insured/unspecified	6.9	8.9	6.6	

Compared to urban children, rural children were more likely to be non‐Hispanic white (74.4% vs 50.1%, *P* < .0001), reside in a household living below the FPL (23.0% vs 18.6%, *P* < .0001), and have public health insurance (35.0% vs 28.0%, *P* < .0001, Table 2). A larger proportion of urban children did not speak English as the primary language in their home, compared to rural children (14.4% vs 6.3%, *P* < .0001). Compared to urban children, a lower percentage of rural children resided with a caregiver with a college education or more (65.0% vs 72.5%, *P* < .0001) and lived in a household with 2 parents, currently married, than their urban counterparts (64.9% vs 67.0%, *P* = .0002).

### Prevalence of PCEs by rural/urban

A higher percentage of rural children reported engaging with a guiding mentor than urban children (94.6% vs 89.0%, *P* < .01; Table 3). Compared to urban children, rural children were less likely to participate in after‐school activities (76.6% vs 80.1%, *P* < .01), and more likely to volunteer (48.0% vs 43.4%, *P* < .01). A larger proportion of rural children reported living in a safe neighborhood than urban children (97.2% vs 94.5%, *P* < .01), as well as living in a supportive neighborhood (59.8% vs 56.3%, *P* < .01). There was no significant difference between rural and urban children for resilient family and connected caregiver, but both values were above 90%.

**TABLE 3:** Positive childhood experiences among children ages 6‐17, National Survey of Children's Health, in total and stratified by residence

Characteristic	All (%)	Rural %	Urban %	*P* value
After‐school activities	79.6	76.6	80.1	.0003
Community volunteer	43.9	48.0	43.4	<.0001
Guiding mentor	89.7	94.6	89.0	<.0001
Connected caregiver	95.6	95.6	95.6	.9212
Safe neighborhood	94.8	97.2	94.5	<.0001
Supportive neighborhood	56.7	59.8	56.3	.0007
Resilient family	92.3	92.1	92.3	.8252

### Multivariable regression results

In adjusted analysis, adjusting for sex, age, race/ethnicity, and special health care needs of the child, as well as caregiver characteristics of relation, language in the home, guardian education, family structure, poverty/income level, and health insurance, rural children remained more likely than urban children to be reported as having PCEs: volunteering in their community (model 2: aOR 1.29; 95% CI 1.18‐1.42), having a guiding mentor (model 3: aOR 1.75; 95% CI 1.45‐2.10), residing in a safe neighborhood (model 4: aOR 1.97; 95% CI 1.54‐2.53), and residing in a supportive neighborhood (model 5: aOR 1.10; 95% CI 1.01‐1.20) than urban children (Table 4).

**TABLE 4:** Adjusted analysis, factors associated with positive childhood experiences among children aged 6‐17, 2017‐2018 National Survey of Children's Health survey

	Model 1 *After‐school activities*		Model 2 *Community volunteer*		Model 3 *Guiding mentor*		Model 4 *Safe neighborhood*		Model 5 *Supportive neighborhood*	
Variable	Point estimate	95% CI^a^	Point estimate	95% CI^a^	Point estimate	95% CI^a^	Point estimate	95% CI^a^	Point estimate	95% CI^a^
Urban	*Referent*		*Referent*		*Referent*		*Referent*		*Referent*	
Rural	0.93	0.83‐1.04	**1.29**	**1.18‐1.42**	**1.75**	**1.45‐2.10**	**1.97**	**1.54‐2.53**	**1.10**	**1.01‐1.20**
*Characteristics of child*										
Gender of child										
Male	*Referent*		*Referent*		*Referent*		*Referent*		*Referent*	
Female	**1.12**	**1.01‐1.25**	**1.37**	**1.28‐1.48**	**1.23**	**1.07‐1.43**	0.92	0.74‐1.13	0.96	0.89‐1.03
Age of child										
6‐12 years old	**0.78**	**0.70‐0.88**	**0.38**	**0.35‐0.41**	0.95	0.83‐1.10	0.83	0.67‐1.03	1.01	0.94‐1.09
13‐17 years old	*Referent*		*Referent*		*Referent*		*Referent*		*Referent*	
Race/ethnicity of child										
Non‐Hispanic white	*Referent*		*Referent*		*Referent*		*Referent*		*Referent*	
Non‐Hispanic African American	0.90	0.76‐1.07	1.03	0.90‐1.18	**0.52**	**0.42‐0.64**	**0.58**	**0.42‐0.79**	**0.62**	**0.54‐0.70**
Hispanic	0.91	0.78‐1.07	**0.79**	**0.70‐0.89**	**0.49**	**0.40‐0.60**	**0.59**	**0.43‐0.80**	**0.68**	**0.60‐0.76**
NH American Indian/Alaska Native	0.88	0.68‐1.14	0.96	0.80‐1.15	**0.46**	**0.35‐0.59**	**0.50**	**0.31‐0.80**	**0.59**	**0.50‐0.71**
NH Asian/Pacific Islander	0.80	0.44‐1.46	0.95	0.63‐1.43	0.74	0.40‐1.34	0.67	0.33‐1.33	0.82	0.57‐1.18
Other	1.05	0.86‐1.27	0.90	0.79‐1.04	0.69	0.54‐0.87	0.49	0.34‐0.71	0.64	0.56‐0.73
Special health care needs										
Yes	**0.69**	**0.61‐0.78**	**0.82**	**0.75‐0.89**	**0.78**	**0.66‐0.92**	**0.53**	**0.42‐0.66**	**0.66**	**0.61‐0.72**
*Characteristics of parent/household*										
Respondent's relation to child										
Mother	*Referent*		*Referent*		*Referent*		*Referent*		*Referent*	
Father	**0.86**	**0.75‐0.99**	**0.87**	**0.80‐0.95**	**0.63**	**0.53‐0.75**	**1.53**	**1.17‐1.99**	1.02	0.94‐1.12
Other	**0.73**	**0.59‐0.91**	**0.60**	**0.51‐0.70**	1.01	0.73‐1.40	**2.20**	**1.31‐3.70**	1.08	0.92‐1.27
Primary language										
English	*Referent*		*Referent*		*Referent*		*Referent*		*Referent*	
Not English	0.82	0.66‐1.01	0.86	0.72‐1.02	**0.50**	**0.40‐0.63**	0.95	0.65‐1.40	0.86	0.72‐1.02
Guardian education										
Less than high school or high school	**0.44**	**0.39‐0.50**	**0.49**	**0.44‐0.54**	**0.70**	**0.59‐0.84**	**0.74**	**0.58‐0.95**	0.95	0.85‐1.06
Some college or more	*Referent*		*Referent*		*Referent*		*Referent*		*Referent*	
Family structure										
Two parents, currently married	*Referent*		*Referent*		*Referent*		*Referent*		*Referent*	
Two parents, not currently married	0.94	0.76‐1.15	**0.57**	**0.48‐0.68**	0.93	0.71‐1.21	**0.57**	**0.40‐0.80**	**0.74**	**0.63‐0.87**
Single mother	1.06	0.91‐1.23	**0.82**	**0.73‐0.91**	0.78	0.63‐0.96	0.81	0.62‐1.06	**0.75**	**0.68‐0.84**
Other	1.07	0.81‐1.42	0.88	0.71‐1.08	0.76	0.50‐1.14	0.76	0.44‐1.33	0.93	0.75‐1.14
Poverty/income level										
0%‐99% federal poverty level	**0.34**	**0.28‐0.41**	**0.78**	**0.67‐0.91**	**0.71**	**0.54‐0.94**	**0.49**	**0.32‐0.75**	**0.62**	**0.53‐0.71**
100%‐199% federal poverty level	**0.41**	**0.35‐0.49**	**0.83**	**0.74‐0.94**	**0.65**	**0.51‐0.83**	**0.54**	**0.36‐0.81**	**0.59**	**0.53‐0.67**
200%‐399% federal poverty level	**0.47**	**0.41‐0.54**	**0.86**	**0.79‐0.93**	**0.78**	**0.64‐0.95**	**0.67**	**0.47‐0.94**	**0.69**	**0.63‐0.75**
400% federal poverty level or above	*Referent*		*Referent*		*Referent*		*Referent*		*Referent*	
Health insurance										
Public	**0.62**	**0.53‐0.72**	**0.79**	**0.70‐0.90**	0.87	0.71‐1.08	**0.48**	**0.36‐0.63**	**0.80**	**0.71‐0.90**
Private	*Referent*		*Referent*		*Referent*		*Referent*		*Referent*	
Public and private	**0.55**	**0.43‐0.71**	**0.71**	**0.58‐0.88**	0.76	0.53‐1.09	**0.53**	**0.34‐0.82**	**0.78**	**0.64‐0.95**
Not insured/unspecified	**0.55**	**0.44‐0.69**	1.07	0.89‐1.30	0.82	0.60‐1.14	**0.48**	**0.30‐0.75**	0.88	0.72‐1.07

*Note*: Bold indicates significance.

^a^ 95% CI = 95% Wald confidence intervals.

Compared to male children, female children were more likely to participate in after‐school activities (model 1: aOR 1.12; 95% CI 1.01‐1.25), volunteer in the community (model 2: aOR 1.37; 95% CI 1.28‐1.48), and have a guiding mentor (model 3: aOR 1.23; 1.07‐1.43). Children ages 6‐12 years of age had a lower odds of participating in after‐school activities (model 1: aOR 0.78; 95% CI 0.70‐0.88) and volunteering in their community (model 2: aOR 0.38; 95% CI 0.35‐0.41) than children 13‐17 years old.

Non‐Hispanic Black children, Hispanic children, and non‐Hispanic American Indian/Alaska Native children were all less likely to have a guiding mentor, reside in a safe neighborhood, or reside in a supportive neighborhood than white children. Children with special health care needs were less likely to experience each of the PCEs modeled, compared to children with no special health care needs. Compared to children with a caregiver with a college education or more, children with a caregiver with a high school education or less were less likely to experience each type of PCE, except for residing in a supportive neighborhood.

Children with 2 parents, not currently married, had a lower likelihood of experiencing a guiding mentor, living in a safe neighborhood, or supportive neighborhood compared to children with 2 parents, currently married. Across all models, children in all FPLs had lower odds of experiencing each type of PCE than children residing at 400% or above the FPL. Compared to children with private insurance, children with public insurance had a lower likelihood of experiencing all PCEs, except for guiding mentors.

## DISCUSSION

This is the first study to use the full NSCH, with all 50 states and the District of Columbia, to examine rural‐urban differences in PCEs using multivariable analysis. Prior research had used 2017‐2018 public use NSCH, which had residence information for 31 states and the District of Columbia, to examine rural‐urban differences in PCES, finding that rural children were more likely to volunteer in their church, school, or community, and more likely to have a mentor for advice or guidance, in adjusted analyses.^8^ Our findings confirm and expand upon this prior work, finding that rural children were more likely to volunteer and have a mentor, but they were also more likely to live in a safe neighborhood and live in supportive neighborhood, which was not found in prior work.

The positive findings from this study are important to disseminate as they may improve the mental health of all children, particularly rural children, and inform the need to continue learning about differences among environmental settings and specific interventions that meet the needs of rural versus urban communities. Rural children face higher rates of many ACEs, which have been shown to increase the likelihood of poorer mental health due to toxic stress.^1^, ^4^ But the multivariable logistic regression findings from this study also demonstrate that rural children have assets in their community which may help to moderate or mitigate ACE prevention—such as the higher likelihood of residing in a safe and supportive neighborhood. The Centers for Disease Control and Prevention (CDC) have discussed safety as a critical component for healthy social‐emotional development in children (CDC). One of the very first priorities listed by the CDC in the *Essentials for Childhood Framework* is safe and stable relationships and environments.^5^

While the findings were overall very positive for rural children and adolescents and their healthy social‐emotional development, there are many compositional characteristics that distinguish rural children from their urban counterparts. In this study, a lower proportion of rural children had a caregiver with at least some college, and a higher proportion of rural children living below the FPL and having public insurance, compared to urban children. Children residing in poverty and having public health insurance were less likely to experience each type of PCE in the adjusted analysis. This has important implications for the design and implementation of programs to promote PCEs in rural communities.

Programs that help to develop healthy social and emotional relationships between the caregiver and child include the *Strengthening Families Program*, a program that works on enhancing parental knowledge of child development, child mental health, and supportive relationships.^2^ These programs can be particularly important for families with high ACE counts, as trauma may be intergenerational, and positive parenting programs can help to decrease the likelihood of intergenerational trauma.^18^ Safe, supportive social‐emotional interactions with caregivers can improve both the short‐term and long‐term mental health of children and adolescents.^18^

The promotion of PCEs at the community and systems level can also be beneficial to children and families, particularly those residing in poverty that may need referrals and connections to community support. One program, the *Safe Environment for Every Kid* (SEEK), links families to community support through their primary care provider.^19^ An exciting new program in the works is *Thriving Families, Safer Children*, which has been developed by the Annie E Casey Foundation and Prevent Child Abuse America.^20^ This program works on the community level, developing more equitable community systems to reduce child poverty and improve intergenerational trauma. Programs such as SEEK and Thriving Families are important steps to improving the availability of PCEs for all children, both in rural and urban communities.

Finally, school mental health professionals may be a primary way for children, particularly rural children who have lower access to mental health providers as they may reside in health care professional shortage areas, to receive mental health services and support.^21^, ^22^ School mental health services provide a location to receive services that are fully integrated into the community and thus less likely to be seen as a place of stigma for receiving services. Prior work has shown that school mental health can be an ideal way to address and improve mental health in rural communities.^23^

### Strengths and limitations

There are numerous strengths to this study including that this is the first study to use the full NSCH, with all 50 states and the District of Columbia, to examine rural‐urban differences in PCEs using multivariable analysis. Limitations of the study include the use of the NSCH which uses an addressed‐based sampling plan, thus not including homeless or transient populations. Further, the PCEs measured in our study are limited to those collected by the NSCH and may not necessarily capture all PCEs that could be experienced by a child. Caregivers may also overstate PCEs since they are socially desirable events.

## CONCLUSIONS

Improving the mental health of rural children through the experience of PCEs is 1 avenue to prevent, moderate, or mitigate the experience of ACEs. The assessment of rural‐urban differences in PCEs using the full NSCH is a unique opportunity to quantify exposure to PCEs. Findings from this study can help to support rural stakeholders, such as rural mental health professionals in their work to improve rural child and adolescent mental health. Further, the findings from this study may help policymakers and program developers best determine how to leverage community resources and assets for the maximum benefit of their residents.

## DISCLOSURES

This project was supported by the Health Resources and Services Administration (HRSA) of the US Department of Health and Human Services (HHS) under grant number #U1CRH30539, Rural Health Research Grant Program Cooperative Agreement. The authors declare no competing financial interests.
